# Dexmedetomidine for conscious sedation with colorectal endoscopic submucosal dissection: a prospective double-blind randomized controlled study

**DOI:** 10.1038/s41424-018-0032-5

**Published:** 2018-07-04

**Authors:** Hideaki Kinugasa, Reiji Higashi, Koji Miyahara, Yuki Moritou, Ken Hirao, Tsuneyoshi Ogawa, Masaki Kunihiro, Masahiro Nakagawa

**Affiliations:** 1 Department of Gastroenterology and Hepatology, Hiroshima City Hiroshima Citizens Hospital, Hiroshima, 730-8518 Japan; 2 Department of Gastroenterology and Hepatology, Okayama University Graduate School of Medicine, Dentistry and Pharmaceutical Sciences, Okayama, 700-8558 Japan

## Abstract

**Objective:**

Conscious sedation for colorectal endoscopic submucosal dissection (ESD) has not been standardized, and there are no studies of sedation for colorectal ESD.

**Methods:**

We conducted a prospective double-blind randomized controlled trial to clarify the usefulness of DEX during colorectal ESD. In total 80 patients with colorectal ESD from April 2016 to May 2017 were assigned to the placebo group or the DEX group (40 cases each). The primary outcome was patient satisfaction (visual analogue scale: VAS). Secondary outcomes were evaluated for 13 factors, including patient pain level (VAS), endoscopist satisfaction (VAS), objective patient pain level viewed from the endoscopist’s perspective (VAS), rate of patient response, rate of side effects, etc., from the patient’s and endoscopist’s perspectives.

**Results:**

Patient satisfaction was 8.4 and 9.1 (*P* = 0.018) in the placebo group and the DEX group, respectively. Secondary outcomes of patient pain level, endoscopist satisfaction, objective patient pain level from the endoscopist’s perspective for the placebo and DEX groups were 1.2 and 0.4 (*P* = 0.045), 8.2 and 9.3 (*P* < 0.001), and 1.2 and 0.5 (*P* = 0.002), respectively. All of these were significantly positive results (more comfortable and less pain) in the DEX group. The rate of a patient response was 100% in all cases. The side effects (hypoxia/bradycardia/hypotension) were 0%/0%/0% and 7.5%/7.5%/5% (*P* = 0.030). However, these rates were less than the reported side effect occurrence rate, and no additional medication was needed.

**Conclusion:**

DEX enables conscious sedation, and is useful not only for patient and endoscopist satisfaction but also for pain relief. DEX is an effective sedation method for colorectal ESD.

## Introduction

Endoscopic submucosal dissection (ESD) is an endoscopic resection technique and a good option for lesions suspected to be superficial neoplasia. Its high en block resection rate allows for precise histological analysis and low recurrence rates^1–3^. With the widespread use of the ESD procedure, the importance of anesthesia is increasing, and the kind of sedation is becoming a key for better outcomes of patient satisfaction. ESD of the esophagus and stomach requires deep sedation because the overtube equipment and the long procedure time are uncomfortable and painful compared with regular endoscopy for screening. Recently, several studies have reported that midazolam and propofol were more reasonable for sedation during endoscopic treatments such as esophagus and stomach ESD^4, 5^. However, unlike ESD of the esophagus and stomach, colorectal ESD is not suitable for deep sedation because it requires many changes, such as breath holding and posture conversion, during the endoscopic procedure to support treatment. Therefore, in colorectal ESD is desirable to remove discomfort and pain under conscious sedation. Dexmedetomidine (DEX) is used as a sedative, and is widely used in intensive care units^6^. Several randomized controlled trials have evaluated the efficacy of DEX compared with midazolam and propofol for gastrointestinal endoscopy screening^7, 8^. A meta-analysis shows that DEX is a safe and effective sedative agent for gastrointestinal endoscopy^9^. However, conscious sedation for colorectal ESD has not been standardized, and there are no studies of sedation for colorectal ESD. It can be hypothesized that DEX can provide effective conscious sedation during colorectal ESD. To clarify the usefulness of DEX during colorectal ESD, we conducted a prospective double-blind randomized controlled trial. The purpose of this study was to determine whether patient and endoscopist satisfaction are superior with DEX. To the best of our knowledge, this is the first report to show the usefulness of DEX with colorectal ESD in a prospective study.

## Methods

### Patients

A total of 80 consecutive ESD procedures for 80 colorectal neoplasms (80 patients) were performed at Hiroshima City Hiroshima Citizens Hospital with or without DEX between April 2016 and May 2017. Inclusion criteria were (1) 18 years<age<90 years, (2) a diagnosis of colorectal superficial neoplasia requiring ESD, (3) consciousness state in daily life, and (4) provision of written informed consent regarding study participation. Exclusion criteria were (1) DEX allergy, (2) severe liver disorder (serum aspartate transaminase and serum alanine transaminase >100 IU/l), (3) severe renal failure (serum creatinine >2 mg/dl), (4) severe heart disease (New York Heart Association Class III or IV), and (5) severe lung disease (chronic obstructive pulmonary disease with dependency on oxygen administered by nasal cannula). This study was approved by the Hiroshima City Hiroshima Citizens Hospital Clinical Ethics Committee on Human Experiments in accordance with the Declaration of Helsinki (clinical trial registration number: UMIN 000021769). All patients provided written informed consent prior to enrollment.

### Study design

**Fig. 1:**
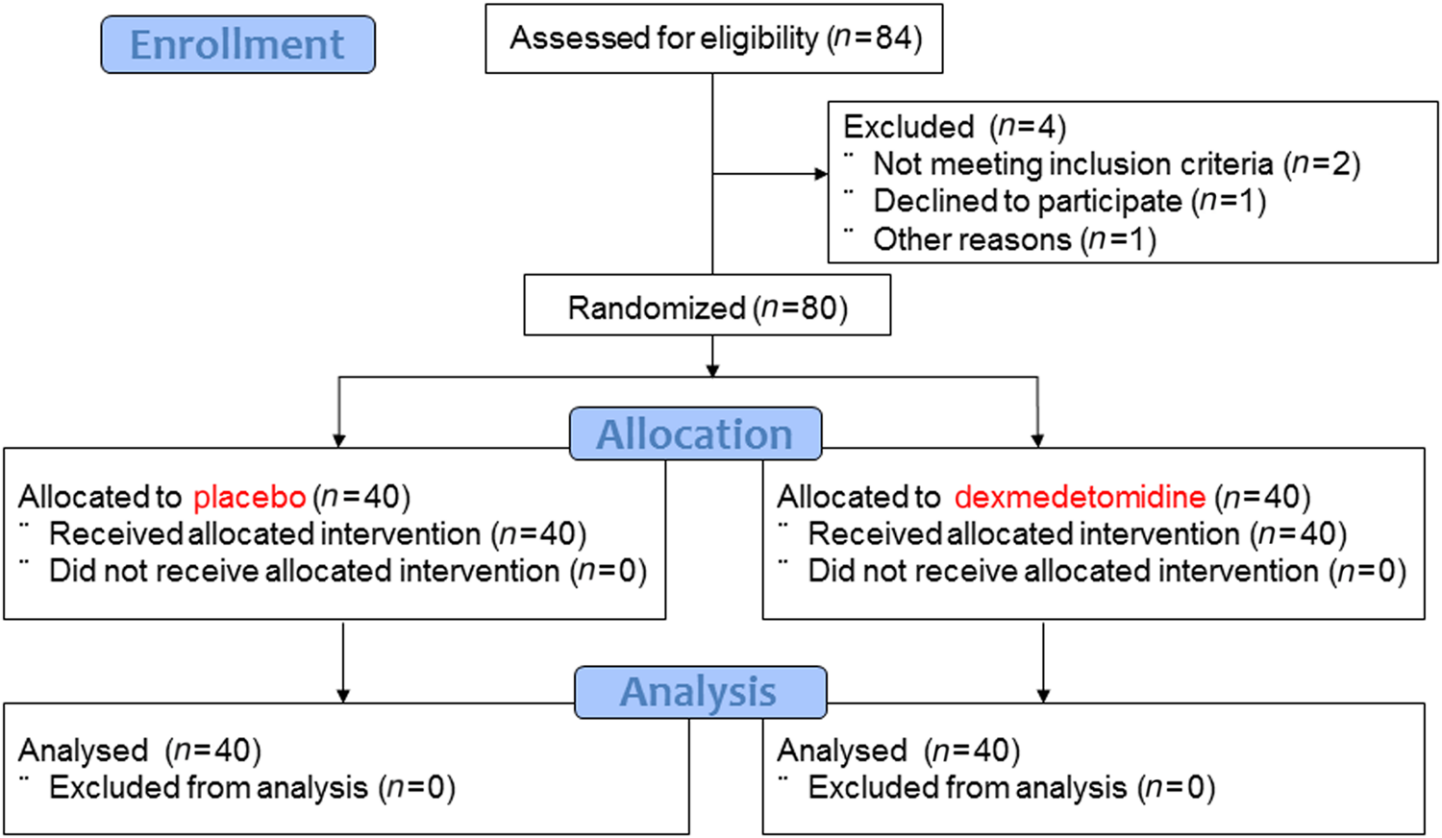
Flowchart of study participants

This study was a double-blind randomized controlled trial at a single center. Patients with colorectal neoplasia scheduled for ESD were included in the study. Patients were randomly assigned to either the placebo group or the DEX group (Fig. 1). Pethidine which is also known as meperidine was used as an analgesic in both groups. The placebo was used as the standard arm in the present study because pethidine is the analgesic most commonly used without sedation during colorectal ESD. The primary outcome was patient satisfaction (visual analogue scale: VAS). Secondary outcomes were patient pain level (VAS), endoscopist satisfaction (VAS), objective patient pain level viewed from the endoscopist’s perspective (VAS), patient movement (VAS), difficulty of the procedure (VAS), rate of patient response (%), rate of en bloc (%), rate of R0 resection (%), resection time (min), resected tumor size (mm), total amount of analgesic (mg), rate of side effects (hypoxia/bradycardia/hypotension) (%), and rate of complications (%). After the procedure, the VAS scores for sedation during the ESD procedure were assessed by the patient and endoscopist using a VAS score sheet. A sample size calculation was based on the VAS score of patient satisfaction to sedation in a preliminary trial (VAS score: 7.5 (75%) in the placebo group (10 patients) and VAS score: 9.5 (95%) in the DEX group (10 patients)). The standard deviation (SD) was 1.5. A power calculation (α = 0.05; β = 0.10) indicated a required total sample size of 74 patients (37 patients with placebo vs. 37 patients with DEX) using a two-tailed *Χ*^2^ test. Projecting a 10% drop-out rate for enrolled patients, the target total sample size was 80 patients (40 patients with placebo vs. 40 patients with DEX) (Fig. 1).

### Medication and monitoring

All medications were administered by nurses who were neither endoscopists nor nurses of the ESD procedures. The nurses had all attended a basic life support (BLS) course. Local pharyngeal anesthesia was performed using 4% lidocaine. The initial infusion of the placebo or DEX as sedation was set at 6.0 μg/kg/h for 5 min and was maintained at 0.4 μg/kg/h. After the initial infusion, an endoscope (PCF-Q260AZI; Olympus Optical Co., Tokyo, Japan.) was inserted. To reach and maintain an adequate level of sedation defined by the Richmond Agitation-Sedation Scale (RASS) between 0 and −3, the titration speed of the placebo or DEX was adjusted by increasing or decreasing by 0.1 μg/kg/h. For analgesia, all patients in both groups received 35 mg of pethidine at the time of induction of sedation and then 17.5 mg of pethidine every 60 min during ESD. As the reversal agent, 0.2 mg of naloxone (Daiichi Sankyo Co., Tokyo, Japan) was administered to both groups after the ESD procedure. Flumazenil (Fuji Pharma Co., Tokyo, Japan) was not administered to both groups as the reversal agent. During the procedure, blood pressure, oxygen saturation, heart rate, and bispectral index (BIS) were continuously monitored and recorded every 5 min using automatic blood pressure monitoring equipment, pulse oximetry, a three-lead electrocardiogram, and BIS monitoring. BIS monitoring is an electroencephalography-based method that measures depth of anesthesia by analyzing the electroencephalogram and uses a complex algorithm to generate an index score, providing an objective measurement of the level of consciousness in sedated patients^10^. The sedation level was checked every 5 min with BIS to maintain not <60 and was assessed every 15 min with RASS to maintain between 0 and −3. Hypotension as a decrease in systolic blood pressure to <80 mmHg, hypoxia as an oxygen saturation <90%, and bradycardia as a pulse rate <40 beats/min were considered adverse events of sedation. RASS is a medical scale used to measure the agitation or sedation level of a patient. The RASS scale, evaluated from −5 (unarousable) to +4 (combative), provides logical feedback with positive numbers representing varying levels of anxiety/agitation and negative numbers representing varying levels of sedation. RASS scale 0 means alert and calm. Conscious sedation is a RASS between 0 and −3^11^.

### ESD procedure

Colorectal ESD is indicated for the treatment of mucosal colorectal neoplasias without submucosal invasion deeper than 1000 μm, when the risk of lymph node metastasis is very low. The initial lesions were classified as having a polypoid growth type or laterally spreading tumor, such as granular type (LST-G) or non-granular type (LST-NG)^12^. Two experienced endoscopists conducted the procedures. The ESD procedure for colorectal neoplasia was performed using a 1.5-mm DualKnife J (KD-655Q; Olympus Optical Co., Tokyo, Japan) for precutting, circumferential mucosal incision, and submucosal resection. Glycerol (10% glycerol and 5% fructose; Chugai Pharmaceutical Co., Tokyo, Japan), MucoUp (0.4% sodium hyaluronate; Johnson & Johnson K.K., Tokyo, Japan) and a small amount of epinephrine and indigo carmine were injected in the mucosal layer to lift the mucosa. High-frequency generators (VIO 300D; ERBE Elektromedizin GmbH, Tübingen, Germany) were used.

All procedures were performed with carbon dioxide insufflation. The total procedure time is defined as the time elapsed from the submucosal injection to the removal of the neoplasia. An en bloc resection was defined as a tumor resection in one piece that included setting a line prior to ESD. Colorectal perforation was defined as a visible hole in the colonic wall that exposed intraperitoneally. Delayed bleeding was defined as bleeding with hematemesis or melena that required endoscopic reintervention or transfusion after the ESD procedure. The degree of submucosal fibrosis was determined based on the findings observed at the time of submucosal dissection and classified into three groups: F0 (no fibrosis), F1 (mild fibrosis), and F2 (severe fibrosis). F0 was defined as a transparent submucosal layer. F1 appeared as a white web-like structure in the transparent submucosal layer, and F2 appeared as a white muscular-like structure without a transparent submucosal layer^13^. Histological diagnoses were based on the Japanese classification of cancer of the colon and rectum^14^, and the Vienna classification^15^. R0 resection (a curative resection) was considered to have been achieved when both the horizontal and vertical margins of the specimen were free of colorectal neoplasia and there was no submucosal invasion deeper than 1000 μm, lymphatic invasion, vascular involvement, or poorly differentiated components^14^.

### Statistical analyses

Continuous variables are reported as the mean ± standard deviation (SD) or the median and interquartile range (IQR), and were compared using Student’s *t*-test for normally distributed variables and the Mann-Whitney U test for non-normally distributed variables. Categorical variables were compared using the *χ*^2^ test and Fisher’s exact probability test. To examine the difference in VAS score between the placebo and DEX groups, the effect of difficulty of the procedure and the interaction between difficulty and DEX was adjusted by the linear regression model. The JMP (version 9.0.0) software packages (SAS Institute, Cary, North Carolina, USA) were used for the analyses and *P* < 0.05 was considered significant.

## Results

### Patient characteristics

There was no difference between the placebo and DEX groups in patient characteristics such as median age, gender (male/female), Body Mass Index (BMI), median tumor diameter, growth type of tumor (Polypoid/LST-G/LST-NG), or tumor occupied lesion (Cecum (C)/Ascending (A)/Transverse (T)/Descending (D)/Sigmoid (S)/Rectum (R)) (Table 1). Also, no significant difference was found in patient backgrounds containing chronic concomitant diseases, such as cardiovascular, neurological, pulmonary, chronic renal failure, hypertension, and diabetes mellitus (Table 1).

**Table 1:** Patient characteristics

Characteristics	Placebo	DEX	*P* value
No. of procedures	40	40	
Median age, year (range)	70 (45–89)	70.5 (49–87)	0.809
Sex, M/F	22/18	19/21	0.502
Median BMI, kg/mm (IQR)	22.1 (20.8–24.4)	22.2 (19.7–25.3)	0.980
Median tumor diameter, mm (IQR)	22(20–35)	28(21.25–32)	0.171
Growth type, no.			0.357
Polypoid	4	2	
LST-G	20	16	
LST-NG	16	22	
Location, no.			0.889
C	8	7	
A	7	8	
T	4	7	
D	3	2	
S	7	8	
R	11	8	
Chronic concomitant diseases, no.			0.420
Cardiovascular	8	3	0.104
Neurological	3	9	0.060
Pulmonary	3	9	0.063
Chronic renal failure	6	3	0.288
Hypertension	9	11	0.605
Diabetes mellitus	4	4	1.000

*DEX* dexmedetomidine, *M/F* male/female, *BMI* body mass index, *IQR* interquartile range, *LST-G* laterally spreading tumor granular type, *LST-NG* laterally spreading tumor non-granular type, *C* Cecum, *A* Ascending; *T* Transverse; *D* Descending, *S* Sigmoid, *R* Rectum

### Primary outcome and secondary outcomes

Patient satisfaction, as the primary outcome, was 8.4 and 9.1 (*P* = 0.018) in the placebo group and DEX group (Table 2), respectively. This was a significantly positive result that DEX could make patients comfortable. The following results were secondary outcomes. Patient pain level from patient’s perception was 1.2 and 0.4 (*P* = 0.045). This was a significantly positive result, as well as a primary outcome, that DEX could remove pain. Endoscopist satisfaction in the placebo and DEX groups was 8.2 and 9.3 (*P* < 0.001), respectively, and objective patient pain level from the endoscopist’s perspective was 1.2 and 0.5 (*P* = 0.002), respectively. There were no significant differences between the two groups for patient movement and difficulty of the procedure, which were endoscopist’s perception VAS score (Table 2). In the placebo and DEX groups, the rate of patient response was 100% (Table 2). There was no significant difference for the rate of en bloc resection, R0 resection, resection time, resected tumor size, or total amount of pethidine. In addition, the rate of side effects (hypoxia/bradycardia/hypotension) were 0% (0/40)/0% (0/40)/0% (0/40) in the placebo group and 7.5% (3/40)/7.5% (3/40)/5% (2/40) in the DEX group (*P* = 0.030) (Table 2). However, these rates were less than the reported side effect occurrence rate and all patients recovered from the adverse events related to sedation with conservative treatment. No additional medication was needed. Regarding complication events related to the procedure, one postoperative bleeding occurred in the DEX group that was successful resolved by endoscopic treatment with Endoclip (Olympus Medical Systems Corp., Tokyo, Japan). No perforation occurred in either group in this study, but one patient had a muscle layer injury in the placebo group (Table 2).

**Table 2:** Primary outcome and secondary outcomes

	Placebo	DEX	*P* value
Patient perception VAS score			
Satisfaction with ESD	8.4 (5.3–9.5)	9.1 (8.1–10.0)	0.018
Pain with ESD	1.2 (0.2–2.9)	0.4 (0–1.7)	0.045
Endoscopist perception VAS score			
Satisfaction with ESD	8.2 (3.7–9.3)	9.3 (8.6–9.8)	<0.001
Objective patient pain	1.2 (0.5–3.8)	0.5 (0.2–1.1)	0.002
Patient movement	0.7 (0.2–1.4)	0.5 (0.1–1.2)	0.309
Difficulty of the procedure	5.4 (1.8–8.3)	2.8 (0.8–7.5)	0.155
Patient response, no. (%)	40 (100)	40 (100)	1.000
En bloc resection, no. (%)	40 (100)	40 (100)	1.000
R0 resection, no. (%)	40 (100)	39 (97.5)	0.314
Median resection time, min (IQR)	86.5 (62.5–127.5)	80 (52.5–150)	0.736
Median resected tumor size, mm (IQR)	30 (25–40)	34 (30–40)	0.205
Median dose of pethidine, mg (IQR)	70 (52.5–70)	70 (52.5–70)	0.963
Side effects			0.030
Hypoxia, no. (%)	0 (0)	3 (7.5)	
Bradycardia, no. (%)	0 (0)	3 (7.5)	
Hypotension, no. (%)	0 (0)	2 (5.0)	
Complications			0.367
Perforation, no. (%)	0 (0)	0 (0)	
Postoperative bleeding, no. (%)	0 (0)	1 (2.5)	

*DEX* dexmedetomidine, *VAS* visual analogue scale, *ESD* endoscopic submucosal dissection, *IQR* interquartile range

### Other outcomes

No significant differences between groups in histology (sessile serrated adenoma/polyp (SSA/P)/adenoma/adenocarcinoma) or fibrosis (F0/F1/F2) were observed (Table 3). RASS in DEX was between 0 and −3; although, in the placebo, it was 0 to −1. Sleeping condition (RASS ≤ −1) in placebo and DEX groups were 6 patients and 33 patients (*P* < 0.001), respectively (Supplementary 1). Two patients who were RASS −3/−4 could be awakened with light stimuli. BIS (at the start of ESD/midway through ESD/at the end of ESD) in placebo and DEX groups were 97/96/98 and 95/88/95 (*P* = 0.001), respectively (Table 3).

**Table 3:** Other outcomes

	Placebo	DEX	*P* value
Histology			0.654
SSA/P, no. (%)	2 (5.0)	4 (10.0)	
Adenoma, no. (%)	17 (42.5)	17 (42.5)	
Adenocarcinoma, no. (%)	21 (52.5)	19 (47.5)	
Fibrosis			0.660
F0	10	13	
F1	25	21	
F2	5	6	
Sleeping during ESD (RASS ≤ −1)	6	33	<0.001
BIS			
At the start of ESD	97(96–98)	95(88–98)	0.005
Midway through ESD	96(94–98)	88(82–97)	0.001
At the end of ESD	98(97–98)	95(82.5–97.5)	0.001

*DEX* dexmedetomidine, *SSA/P* sessile serrated adenoma/polyp, *ESD* endoscopic submucosal dissection, *RASS* Richmond Agitation-Sedation Scale, *BIS* bispectral index

### Subanalysis

**Fig. 2:**
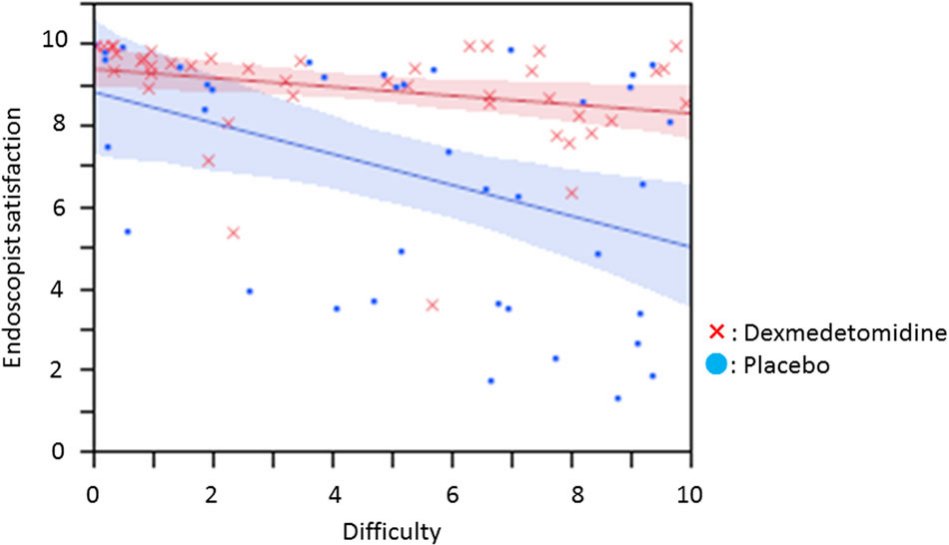
Scatterplots show correlations between endoscopist satisfaction, visual analogue scale (VAS) score, and difficulty of the procedure in patients with the placebo or dexmedetomidine (DEX). Endoscopist satisfaction and VAS score of DEX (red) was significantly higher than that of the placebo (blue), regardless of the difficulty of the procedure. Lines and colored areas indicate regression curves and 95% confidence interval (CI), respectively

A subanalysis of the correlation between DEX and several factors (such as age, sex, tumor size, resection size, resection time, and fibrosis) was examined for satisfaction VAS score and pain VAS score. All factors had some tendency to improve satisfaction and pain with DEX from comparing each median value. A subanalysis of patient satisfaction showed that resection time (83<) was a significant factor with DEX (P = 0.047). Patient pain showed that age (≤70) (*P* = 0.026), resection size (32<) (*P* = 0.049), and resection time (83<) (*P* = 0.017) were significant factors with DEX (Table 4). A subanalysis revealed that DEX affected endoscopist satisfaction strongly regardless of factors such as resection size and resection time (Supplementary 2). On the other hand, DEX did not seem to influence patient movement and difficulty of the procedure. However, the correlation between endoscopist satisfaction and difficulty of the procedure showed that DEX could increase endoscopist satisfaction in higher difficulty cases compared to the placebo group, while endoscopist satisfaction was almost the same level in lower difficulty procedures between the placebo and DEX groups (Fig. 2). A subanalysis to evaluate bias for the two endoscopists was performed for patient satisfaction, patient pain, endoscopist satisfaction, and resection time. There was no significant difference between the two endoscopists for factors.

**Table 4:** Subanalysis for patient satisfaction and pain

Factors for patient satisfaction		Placebo	DEX	Placebo	DEX	*P* value
		*n*	*n*	Median (IQR)	Median (IQR)	
Age	≤70	19	18	8.3 (5.3–9.5)	8.7 (7.9–9.7)	0.162
	70<	21	22	8.5 (5.4–9.6)	9.2 (8.2–10.0)	0.065
Gender	M	22	19	8.4 (6.8–9.5)	9.1 (8.1–9.7)	0.116
	F	18	21	8.5 (3.9–9.8)	9.1 (8.1–10.0)	0.089
Tumor size	≤25	21	14	8.4 (4.3–9.6)	8.9 (8.1–10.0)	0.193
	25<	19	26	8.4 (5.3–9.5)	9.1 (7.9–10.0)	0.073
Resection size	≤32	21	15	8.4 (5.9–9.5)	9.2 (8.1–10.0)	0.088
	32<	19	25	8.3 (5.2–9.7)	9.1 (7.9–10.0)	0.105
Resection time	≤83	19	21	8.4 (6.3–9.8)	9.2 (7.8–10.0)	0.182
	83<	21	19	8.2 (5.2–9.2)	8.8 (8.1–10.0)	0.047
Fibrosis	F0	10	13	8.4 (3.4–9.6)	9.3 (8.6–10.0)	0.092
	F1/F2	30	27	8.4 (6.1–9.5)	8.8 (8.0–10.0)	0.136
Factors for patient pain						
Age	≤70	19	18	2.1 (0.6–5.2)	0.5 (0–2.0)	0.026
	70<	21	22	0.1 (0–1.5)	0.3 (0–1.6)	0.484
Gender	M	22	19	1.0 (0.1–1.9)	0.2 (0–1.7)	0.165
	F	18	21	1.8 (0.4–3.6)	0.6 (0–2.2)	0.174
Tumor size	≤25	21	14	1.1 (0.1–3.9)	0.3 (0–1.4)	0.123
	25<	19	26	1.2 (0.2–2.9)	0.6 (0–1.7)	0.137
Resection size	≤32	21	15	0.7 (0.1–3.9)	0.4 (0–2.8)	0.372
	32<	19	25	1.7 (0.5–2.9)	0.4 (0–1.6)	0.049
Resection time	≤83	19	21	1.0 (0–2.9)	0.4 (0–3.2)	0.555
	83<	21	19	1.3 (0.5–2.9)	0.4 (0–1.0)	0.017
Fibrosis	F0	10	13	1.7 (0.5–5.2)	0.5 (0–3.8)	0.098
	F1/F2	30	27	1.1 (0–2.7)	0.4 (0–1.6)	0.16

*DEX* dexmedetomidine

## Discussion

Deep sedation has been often preferred in esophagus and stomach ESD. However, the suitable sedation level in colorectal ESD is different from esophagus and stomach ESD because the effect of respiratory variation is strong and position conversion is often required during treatment. Conscious sedation is the best for colorectal ESD to increase patient satisfaction, remove patient pain, allow responses during treatment, and promote smooth treatment. The present study revealed that DEX allowed conscious sedation in colorectal ESD. DEX improved not only patient satisfaction but also patient pain and endoscopist satisfaction. Also, DEX enabled patients to hold a breath and change posture during the colorectal ESD procedure. Interestingly, DEX was more effective for cases with long resection times from the view of patient satisfaction, while DEX improved patient pain in young patients, those with large resection sizes and long resection times, and increased endoscopist satisfaction. Another benefit of conscious sedation is that the patient can respond to an order, even in a sedative state. It was possible for all patients with DEX to respond to requests from endoscopists during ESD as well as patients without DEX. No matter how deeply the patients were sleeping, patients could be awaked easily with a call or light stimuli. Fortunately, there were no patients with perforation in this study. However, if occurred, conscious sedation brought positive effect because of its function to relieve pain. Endoscopic treatment with Endoclip will be the first step for treatment even under conscious sedation. DEX, which is an α2-adrenoceptor agonist, has been used frequently to create better sedation. DEX has a neuroprotective effect on the central nervous system through both a direct action on the α2A-adrenoceptor on the impaired nerve and an indirect action via the α2A-adrenoceptor on the astrocyte to develop brain-derived neurotrophic factor^16^. Moreover, DEX has an immunomodulatory action that suppresses the production of cytokines, which leads to a reduced degree of systemic inflammatory responses^16–18^. Using DEX to sedate patients makes it easy to obtain the level of conscious sedation and to remove pain, which means patients are asleep but easy awaken with light stimuli. Our study showed that DEX could remove pain better than we expected. Also, it may prevent inflammation of wounds after ESD with pharmacological action, although the recovery condition of wounds could not be evaluated in this study. With the development of endoscopic examination, the importance of sedation is also increasing. There are many reports on the introduction of sedation in endoscopical procedures. However, side effects and complications are always challenges in sedation. Wernli et al.^19^ reported that the overall risk of complications after colonoscopy increased when individuals received anesthesia service, and the widespread adoption of anesthesia services with colonoscopy should be considered within the context of all potential risks and benefits. The present study showed that the frequency of side effects was lower, and perforation did not occur in the DEX group. After the end of this clinical trial, an additional consecutive 40 patients have had colorectal ESD with DEX at our institution and there have been no adverse events requiring surgery and another medication (data not shown). We speculate that advantages of conscious sedation with DEX may overcome the potential risk. ESD and endoscopic mucosal resection (EMR) are two major techniques for superficial neoplasia. Recently, a meta-analysis for colorectal ESD showed a very low recurrence rate after colorectal ESD (2.0%) at 12 months and a low endoscopic perforation rate (5.2%)^1^. Conversely, a meta-analysis for colorectal EMR showed a recurrence rate after colorectal EMR (13.8%) at 12 months and a low endoscopic perforation rate (1.5%)^20^. Both treatment methods have advantages and disadvantages. However, the cooperation of patients becomes essential to ESD and EMR when endoscopical treatment will be difficult due to size and location. That is why it is advisable to treat with an analgesic without a sedative to avoid deep sedation regardless of the method. Conscious sedation was useful for colorectal ESD in this study, but DEX may also be useful for colorectal EMR. This study has shown that DEX has the possibility of becoming a gold standard in colonoscopical treatment. We should explore both techniques for anesthesia, ESD or EMR-related devices, and SM injection fluids to make ESD/EMR easier to perform. Our study has some limitations. First, this was a single-center trial. A multicenter trial is needed to enhance the results. Second, the sample size was 80, and the primary outcome could be proved but a subanalysis might be warranted for other findings if there were more samples. Due to limitation number of patients included in this study, precise rate of adverse event should be measured in the next trial. In conclusion, DEX enables conscious sedation, and is useful not only for patient and endoscopist satisfaction, but also for pain relief. DEX is an outstanding sedation method for colorectal ESD.

## Study Highlights

### What is current knowledge

- With the development of endoscopy, the importance of sedation is increasing.
- Conscious sedation for colorectal endoscopic submucosal dissection (ESD) has not been standardized.

### What is new here

- Dexmedetomidine (DEX) was useful for conscious sedation with colorectal ESD.
- DEX improved not only patient satisfaction, but also patient pain and endoscopist satisfaction.

## Electronic supplementary material

Supplementary Tables
