# Supplementary material for: Dexmedetomidine for conscious sedation with colorectal endoscopic submucosal dissection: a prospective double-blind randomized controlled study
Source: Clin Transl Gastroenterol. 2018 Jul 4;9(7):167. doi: 10.1038/s41424-018-0032-5 (PMC6030068; doi:10.1038/s41424-018-0032-5)
Supplement: Supplementary file 1 — Supplementary Tables [file 41424_2018_32_MOESM1_ESM.docx]

Supplement 1. RASS score

| RASS score | Condition | Placebo | DEX |
| --- | --- | --- | --- |
| 4 | Combative | 0 | 0 |
| 3 | Very agitate | 0 | 0 |
| 2 | Agitated | 0 | 0 |
| 1 | Restless | 0 | 0 |
| 0 | Alert and calm | 34 | 7 |
| -1 | Drowsy | 6 | 26 |
| -2 | Light sedation | 0 | 5 |
| -3 | Moderate sedation | 0 | 2 |
| -4 | Deep sedation | 0 | 0 |
| -5 | Un-rousable | 0 | 0 |

Abbreviations: DEX, dexmedetomidine; RASS, Richmond Agitation-Sedation Scale.

Supplement 2. Subanalysis for endoscopist satisfaction

|  | | Placebo | DEX | Placebo | DEX |  |
| --- | --- | --- | --- | --- | --- | --- |
| Factors for endoscopist satisfaction | | n | n | Median (IQR) | Median (IQR) | P value |
| Age | ≤70 | 19 | 18 | 8.1 (3.6-9.2) | 8.9 (8.1-9.5) | 0.065 |
|  | 70< | 21 | 22 | 8.4 (3.8-9.4) | 9.5 (8.9-9.9) | 0.003 |
| Gender | M | 22 | 19 | 7.8 (4.6-9.1) | 9.5 (8.7-9.8) | 0.002 |
|  | F | 18 | 21 | 9.0 (3.5-9.4) | 9.3 (8.0-9.6) | 0.085 |
| Tumor size | ≤25 | 21 | 14 | 9.0 (4.4-9.5) | 9.2 (8.7-9.9) | 0.125 |
|  | 25< | 19 | 26 | 6.4 (3.6-8.9) | 9.4 (8.1-9.7) | <0.001 |
| Resection size | ≤32 | 21 | 15 | 9.0 (4.3-9.5) | 9.4 (8.7-9.8) | 0.038 |
|  | 32< | 19 | 25 | 6.4 (3.6-8.9) | 9.3 (8.0-9.7) | 0.001 |
| Resection time | ≤83 | 19 | 21 | 9.0 (3.7-9.6) | 9.5 (8.8-9.8) | 0.038 |
|  | 83< | 21 | 19 | 7.4 (4.2-9.1) | 9.0 (8.1-9.6) | 0.006 |
| Fibrosis | F0 | 10 | 13 | 7.1 (3.6-9.7) | 9.4 (9.0-9.8) | 0.144 |
|  | F1/F2 | 30 | 27 | 8.2 (4.6-9.2) | 9.1 (8.2-9.7) | 0.004 |

Abbreviations: DEX, dexmedetomidine.
